# Vehicle routing optimization and algorithms for instant delivery under customer loss mechanism

**DOI:** 10.1371/journal.pone.0345043

**Published:** 2026-07-27

**Authors:** Gaoming Cao, Weixiong Zha

**Affiliations:** 1 School of Transportation Engineering, East China Jiaotong University, Nanchang, Jiangxi, China; 2 Academic Center, Jiangxi Federation of Social Science Circles, Nanchang, Jiangxi, China; Beijing University of Technology, CHINA

## Abstract

In the field of instant delivery, the mismatch between delivery resources and customer demands has led to increasingly significant customer losses. To address this issue, this study introduces the customer loss mechanism and constructs an evaluation function to screen out resource-intensive customers, thereby clarifying the scope of delivery services. Based on this, this study establishes the vehicle routing optimization model under the customer loss mechanism with the objective of minimizing the sum of vehicle fixed costs, variable routing costs, and time window penalty costs. An improved genetic algorithm is employed to solve this model. Case study results demonstrate that the improved genetic algorithm outperforms traditional genetic algorithms and tabu search algorithms in convergence speed, optimization capabilities, and stability, reducing total delivery cost by 36.25% and 4.18%, respectively, with zero delivery violations. Regarding model performance, when proactively excluding 8.33% of customers, the total delivery cost is reduced by 17.18%, primarily driven by the reduction in fleet size. Furthermore, large-scale experiments reveal a pronounced leverage effect: excluding a mere 5% of marginal customers counter-intuitively reduces both fleet size and travel distance, while a 10% loss yields an 18.39% total delivery cost reduction with zero violations, proving that the mechanism precisely screens out inefficient nodes rather than arbitrarily rejecting them. Sensitivity analysis further confirms the model’s robustness across varying resource tightness, demonstrating that proactive customer loss is a feasible and effective strategy for improving resource utilization through precise resource focusing.

## 1 Introduction

With the development of e-commerce and the advancement of digital life, instant delivery has gained wide popularity among consumers by satisfying their on-demand immediate fulfillment needs, and its market size has also expanded rapidly.

However, behind such rapid industry expansion, the problem of customer loss caused by the mismatch between instant delivery resources and customer demands has become increasingly prominent. According to the 2025 Instant Retail Industry In-depth Report by China Merchants Securities, approximately 13% of consumers directly give up their purchases due to the lack of timely and effective delivery services. This delay-induced customer loss is particularly severe during peak order periods. Against this backdrop, instant delivery platforms should move beyond passively enduring customer loss. Instead, they ought to proactively screen and exclude customers with poor resource-demand matching, and adopt a selective service strategy. This approach enables focused resource allocation to efficiently serve customers within the feasible service scope, reduces delivery violation rates, and ultimately achieves the dual goals of stabilizing and expanding market share.

As an important variant of the Vehicle Routing Problem (VRP), the instant delivery routing problem has garnered existing research that mainly focuses on the trade-off between delivery cost and efficiency. For example, Voigt et al. [1] proposed offering slower delivery options for certain demands, while Chen et al. [2] explored leveraging emerging technologies to accelerate online scheduling.

Furthermore, considering heterogeneous customer demands and their impact on costs has gained attention, with studies analyzing purchasing behaviors [3] and preferences for autonomous vehicles [4]. In particular, time-sensitive customer preferences and their cost implications have also been examined [5].

In terms of enhancing efficiency through delivery methods, researchers have looked into joint operations combining ground vehicles with drones [6,7]. Finally, addressing network constraints and timeliness has led to dynamic routing optimizations under congestion [8] and innovative order-splitting strategies [9].

In summary, although existing research on instant delivery vehicle routing spans various dimensions—such as costs, preferences, delivery modes, and road networks—the overarching goal remains consistent: striking a balance between total delivery cost and efficiency, provided that all customer demands are fulfilled.

To resolve the core dilemma arising from mismatched delivery resources and customer demands, current studies largely adopt methods like differentiated services with priority mechanisms, and resource expansion with flexible scheduling.

Regarding the first approach of differentiated services with priority mechanisms, Kovacs et al. [10] argued that during vehicle delivery, customer segmentation should be combined with cost-benefit analysis.

Specifically, implementation strategies include setting different time window penalty costs based on customer value classifications [11,12] and providing services in priority order based on customer prioritie [13,14].

Although prioritizing certain customers may increase total delivery costs [15,16], total delivery costs typically decrease as the flexibility of customer priority rules increases [17]. Conversely, routes without prioritized customers tend to exhibit higher average vehicle capacity utilization [15].

Regarding the second approach of resource expansion and flexible scheduling, existing research primarily highlights the deployment of forward warehouses. By integrating storage and delivery functions, forward warehouses enable nearby dispatching, which effectively alleviates resource constraints. This spatial proximity significantly shortens delivery times and improves fulfillment efficiency [18], thereby enhancing overall customer satisfaction [19].

To fully exploit this expanded capacity, modeling efforts have evolved from basic routing to complex network and joint scheduling designs. For instance, Chen et al. [20] incorporated realistic factors like traffic congestion and carbon emissions into a cold chain routing model under the forward warehouse mode. At the network level, Guo et al. [21] constructed a two-echelon structure (urban central warehouse – forward warehouse – customer) to optimize multi-trip routing with time windows for fresh e-commerce. Furthermore, breaking down operational silos, Chen et al. [22] developed a mixed-integer model that jointly optimizes zone picking and vehicle routing with time windows within the forward warehouse system.

Beyond service differentiation and resource expansion, optimizing delivery methods serves as another crucial lever for enhancing efficiency. To address traditional delivery challenges, autonomous tools like drones and robots are widely recognized for their potential to transform last-mile logistics [23].

Studies consistently show that integrating these autonomous systems with traditional fleets or public transport significantly reduces costs, mitigates traffic uncertainties, and ensures on-time performance [24–27].

Furthermore, crowdsourcing models offer a flexible capacity supplement to alleviate resource strain. Research in this area primarily focuses on coordinating dual time windows between customers and drivers [28], integrating heterogeneous outsourced capacities into sustainable networks [29], and tailoring order allocations based on individual driver behaviors [30].

The above review indicates that while existing studies have developed multi-dimensional strategies to address the core dilemma of resource-demand mismatch, they remain largely confined to a “deliver-all” paradigm. A critical reality has been overlooked: regardless of capacity expansion, facility densification, or model innovation, the hard constraint of demand outstripping resources remains unavoidable during peak hours (e.g., meal times) or unforeseen disruptions (e.g., severe weather). Consequently, the system inevitably faces the dilemma of failing to fulfill all orders. Yet, existing research remains predominantly passive in accommodating customer demands, paying insufficient attention to scenarios where demand outstrips service capacity; that is, the critical issue of strategically excluding certain orders under hard resource constraints to safeguard core services remains largely unexplored.

Given that full-demand coverage approaches cannot fundamentally resolve the core contradiction between delivery resources and customer demand, selective service strategies have become a key research focus in recent literature. Regarding selective service strategies, initial studies focused on static customer classification. For instance, Stavropoulou et al. [31] categorized customers into “must-visit high-frequency” and “non-mandatory low-frequency” groups, suggesting that the latter should only be served using surplus capacity to maximize VRP profits. Under stricter resource constraints, Akkerman et al. [32] noted that in resource-constrained scenarios, prioritizing services for closer customers with higher demand directly improves delivery efficiency and resource utilization. Extending this to dynamic environments, Giallombardo et al. [33] emphasized the need to balance “immediate returns” against “reserving capacity for higher-value future requests.”

Overall, current studies on selective delivery primarily operate under a premise of capacity surplus, aiming to maximize benefits by serving optional customers with excess resources. Consequently, they largely neglect how to cope with scenarios of actual resource scarcity.

In light of the persistent resource-demand mismatch, existing literature—whether confined to a “deliver-all” paradigm or reliant on a “surplus-capacity” premise—fundamentally fails to resolve the dilemma of proactively filtering demands under rigid resource constraints. Addressing this, our study embraces the reality of capacity scarcity and formulates the instant delivery routing problem as a new variant of the Vehicle Routing Problem, which we call the Vehicle Routing Problem under Customer Loss Mechanism (VRP-CLM). This model drives a paradigm shift in two ways: it explicitly formulates service acceptance as a binary decision variable, granting the system the authority to actively reject orders; and it front-loads the customer screening process prior to route construction. Formulated to minimize the total delivery costs—comprising vehicle fixed costs, variable routing costs, and time window penalty costs—the model is solved using an Improved Genetic Algorithm (IGA). Numerical results verify that by strategically sacrificing a marginal fraction of high-cost orders, this approach significantly reduces total delivery costs while enhancing system resilience and resource efficiency.

The primary contributions of this study lie in three interrelated aspects.

First, we drive a paradigm shift from passive customer loss to proactive demand screening. Recognizing the persistent mismatch between capacity and demand, we introduce the customer loss mechanism. By developing a customer loss mechanism evaluation function via the AHP-entropy method, we provide a scientific basis for instant delivery platforms to transition from a rigid “deliver-all” approach to a flexible “selective coverage” strategy, thereby safeguarding core service quality and resource efficiency.

Second, we construct a novel VRP-CLM model that fundamentally alters the traditional routing process. Its core innovation lies in front-loading the decision timeline—explicitly formulating service acceptance as a binary variable enables early-stage customer screening. This structural change prevents the resource fragmentation and cascading failures inherent in forced full-coverage models.

Third, we design the IGA incorporating hybrid evolutionary strategies to efficiently solve the proposed model. We rigorously validate both the algorithm’s computational superiority and the model’s practical value through benchmarking against Traditional Genetic Algorithms (TGA) and Tabu Search (TS) across empirical case studies.

The remainder of this paper proceeds as follows. The second chapter elaborates on the customer loss mechanism. The third chapter establishes the mathematical model VRP-CLM. The fourth chapter presents the detailed design of the IGA algorithm. The fifth chapter sets up numerical experiments and analyzes corresponding computational results. The final chapter summarizes this study and puts forward directions for future research.

## 2 Mechanism, influencing factors, and evaluation function

### 2.1 Formation mechanism of the customer loss mechanism

The customer loss mechanism is fundamentally a resource-demand balancing mechanism designed to sustain core operational stability under rigid resource constraints in instant delivery systems. Specifically, it comprises three key components:

#### 2.1.1 System imbalance triggering mechanism.

When total customer demand exceeds the fulfillment capacity of existing delivery resources (e.g., when vehicle capacity is saturated or time window constraints are tight), the system activates an imbalance alert. At this juncture, customer requests must be systematically evaluated, and certain requests proactively excluded, thereby enabling the system to rapidly recover from an overloaded state back to a balanced state.

#### 2.1.2 Trade-off decision-making.

The selection of excluded customers is not arbitrary; rather, it relies on a quantitative evaluation framework based on multi-dimensional indicators. During this process, factors such as customer value and time window flexibility are comprehensively assessed to quantify the cost of customer loss. This quantitative model not only eliminates subjective biases but also ensures the consistency and reproducibility of decision criteria across various operational scenarios.

#### 2.1.3 Resource reallocation.

By narrowing the service scope, the platform releases critical resources (e.g., vehicles capacity) and reallocates them to retained customers. This targeted reallocation not only avoids breaches in instant delivery commitments but also reduces the overall delivery costs through route consolidation and precise time window management.

### 2.2 Selection of factors influencing customer loss

Instant delivery involves core processes such as timeliness assurance, cost control, and resource allocation. These processes are interconnected and mutually influential, collectively determining the overall service performance of instant delivery systems.

#### 2.2.1 Customer value ($c_{ve}$).

Customer Relationship Management theory and Pareto’s law suggest that under resource constraints, enterprises should prioritize allocating resources to high-value customers. Such prioritization helps achieve efficient resource utilization and profit maximization. For low-value customers, appropriate cuts in resource investment can be considered to prevent operational inefficiency caused by resource misallocation. Multiple approaches are available for customer value classification, including the Recency-Frequency-Monetary model [34] and the Activity Based Classification method [35]. This study adheres to the principle of customer value prioritization, with the goal of directing limited resources to serve a small number of key customers first and thus enhancing overall resource allocation efficiency.

#### 2.2.2 Customer time window width ($c_{tw}$).

In the context of instant delivery, customer time window width can be measured by the width of the time window: a narrower window implies lower customer tolerance for delivery time flexibility and thus higher urgency. The width of the time window directly determines the risk of a contract breach. Narrower time windows lead to higher explicit costs, such as penalties for delays and customer complaints. Assuming the start time of customer *i*’s time window $e_{i}$ is and the end time is $l_{i}$, the width of the time window is calculated as $l_{i}-e_{i}$. This study follows the principle of prioritizing customers with wide time windows. By treating customers with loose time windows as high-priority service objects and strategically excluding those with tight deadlines, the platform can effectively reduce time window penalty costs.

#### 2.2.3 Customer Distance from the Depot ($c_{dd}$).

Customer distance from the depot exhibits a linear positive correlation with delivery costs. Additionally, customers located farther away may lead to route detours and extend the total delivery duration. Euclidean distances between the distribution depot and customers, as well as between customer nodes, can usually be obtained using Geographic Information Systems and similar technologies.

Furthermore, considering actual road conditions, the straight-line distance can be adjusted using map data and algorithms to better reflects the real driving distance. For example, a road curvature coefficient σ can be introduced. Let $\left(x_{i},y_{i}\right)$ and $\left(x_{j},y_{j}\right)$ represent the coordinates of points *i* and *j*, respectively. The straight-line distance between points *i* and *j* is calculated as $d_{ij}=\sqrt{(x_{i}-x_{j}{)}^{2}+(y_{i}-y_{j}{)}^{2}}$, and the actual driving distance is $c_{dd}=\sigma \cdot d_{ij}$. This study assumes that the road curvature coefficient σ is 1, meaning regional differences in road conditions are not considered. It follows the principle of prioritizing customers by their proximity to the distribution depot (i.e., serving closer customers first).

#### 2.2.4 Customer demand volume ($c_{dv}$).

Demand volume directly impacts vehicle loading rates: small-batch orders tend to cause waste vehicle space and increase unit delivery costs, while large-volume orders can improve resource utilization efficiency through centralized delivery.

In general, when measuring customer demand volume, metrics such as the number of items, weight, or volume in a customer’s order can be used. However, due to the wide variety of goods, significant differences in attributes (e.g., volume) across different products may complicate subsequent calculations and comparisons. To address this issue, the demand volume of various goods can be uniformly converted into standard demand volume (measured in weight or volume) based on standard conversion factors between product volume and weight. This ensures data consistency and comparability, facilitating subsequent analysis and processing. In this study, the volume of customer demand orders is adopted as the metric for customer demand volume. The study follows the principle that, under resources are scarcity, customers with larger demand volume are prioritized to improve resource utilization efficiency.

The four key influencing factors outlined above comprehensively cover multiple critical dimensions of instant delivery. Theoretically, $c_{ve}$, $c_{tw}$, $c_{dd}$, and $c_{dv}$ respectively reflect customer contribution, delivery timeliness, cost risk, and resource allocation efficiency. These factors are mutually independent yet closely interrelated, forming a complete customer loss evaluation system.

In practical application: $c_{ve}$ shows a significant negative correlation with customer loss rate, indicating that losing high-value customers has a greater impact on enterprises; $c_{tw}$ is strongly negatively correlated with customer complaint rates, underscoring that wider time windows reduce the risk of complaints and the importance of on-time delivery for customer satisfaction; $c_{dd}$ exhibits a strong positive correlation with delivery costs, highlighting the critical role of distance in cost control; $c_{dv}$ is closely associated with vehicle loading rates and delivery efficiency, reflecting its significance for rational resource allocation.

### 2.3 Construction of the customer loss mechanism evaluation function

In the VRP-CLM, the customer loss mechanism evaluation function plays a critical preliminary role: its calculation results directly determine the final set of customers to be included in delivery services. Based on the above analysis of factors influencing customer loss, the preliminary expression of the customer loss mechanism evaluation function $L_{i}$ is constructed as follows:


$$\begin{array}{c} L_{i}=f\left(c_{ve}(i),\;c_{tw}(i),\;c_{dd}(i),\;c_{dv}(i)\right) \end{array}$$ (1)

In Equation (1), $L_{i}$ denotes the customer loss value of customer *i*, while $c_{ve}$, $c_{tw}$, $c_{dd}$, and $c_{dv}$ represent customer value, time window width, distance from the depot, and demand volume respectively.

Once the customer loss values are computed, this study determines the customer loss threshold θ using a predefined allowed loss proportion, balancing computational efficiency with adaptability to real-world instant delivery scenarios. This proportional approach enables the platform to directly control the scale of excluded demands, facilitating rapid alignment between remaining orders and rigid delivery capacities. Specifically, the values for all customers are sorted in ascending order, and the threshold θ is set at the quantile corresponding to the target allowed loss proportion—effectively, this excludes a predetermined proportion of customers with the lowest loss scores.

Customers with loss values below this threshold—indicating the lowest cost of loss—are excluded from the current service scope, whereas those above it are retained. Mathematically, customer *i* is discarded if $L_{i}<\theta$, and retained if $L_{i}\geq \theta$. This ensures that the platform strategically filters out the least costly demands, thereby minimizing overall corporate losses.

## 3 Model construction

### 3.1 Problem description

The VRP-CLM can be described as follows: An instant delivery center services a set of customers, and its delivery network is represented as a graph *G* = (*V*, *E*). Here, the node set *V* consists of a single distribution depot (denoted as node 0) and a set of customer nodes N={1,2,…,n}; the edge set E={(i,j)∣i,j∈V} represents all possible travel routes between nodes. The delivery center uses a fleet of vehicles denoted by set *K*, K={k∣k=1,2,…,m}, and each vehicle has a maximum loading capacity of *Q*. When delivery resources fail to match customer demands, the delivery center first evaluates each customer based on specific service requirements and determines the retained customer set $N^{\prime }$ ($N^{\prime }\subseteq N$) using the customer loss mechanism evaluation function. It then generates optimal vehicle delivery routes accordingly. The objective of the proposed model is to minimize the total delivery cost, which comprises three components: vehicle fixed costs, variable routing costs, and time window penalty costs.

### 3.2 Model assumptions

To formulate the problem, the following assumptions are adopted for this model:

1. The locations of the distribution depot and customer points, as well as customer demand volumes, are deterministic and known;
2. The demand volume of each customer does not exceed the maximum load capacity of a single vehicle, and order splitting is not allowed;
3. Each vehicle can serve multiple customers, while each customer is served by exactly one vehicle;
4. All vehicles depart from the distribution center, serve customers along a predefined route, and finally return to the distribution center;
5. The customer loss values remain unchanged during the entire delivery process.

### 3.3 Model construction

#### 3.3.1 Relevant parameters and decision variables.

All relevant parameters and decision variables used in the proposed model are defined in Table 1.

**Table 1:** Definitions of parameters and decision variables.

Symbol	Description
**Parameters**	
*f*	Vehicle fixed cost per vehicle
*c*	Variable routing cost per unit distance
*v*	Vehicle travel speed
$a_{i}$	Actual arrival time of the vehicle at customer *i*
$e_{i}$	Earliest allowed arrival time at customer *i*
$l_{i}$	Latest allowed arrival time at customer *i*
$t_{ij}$	Travel time of the vehicle between customer *i* and customer *j*
$s_{i}$	Service time of the vehicle at customer *i*
$q_{i}$	Demand volume at customer *i*
$d_{ij}$	Distance between node *i* and node *j*
$L_{i}$	Customer loss value of customer *i*
**Decision variables**	
$y_{i}$	0-1 variable; $y_{i}=1$ if customer *i* is included in the service, else 0
$u_{k}$	0-1 variable; $u_{k}=1$ if vehicle *k* is activated, else 0
$x_{ij}^{k}$	0-1 variable; $x_{ij}^{k}=1$ if vehicle *k* travels from *i* to *j*, else 0

#### 3.3.2 Objective function.

The objective of this model is to minimize the total delivery cost. The specific formulation is as follows:


$$\begin{array}{c} \mathrm{Min}T_{c}=f\cdot \sum_{k=1}^{m}u_{k}+c\cdot \sum_{k=1}^{m}\sum_{i=0}^{n}\sum_{j=0}^{n}d_{ij}\cdot x_{ij}^{k}+\sum_{i=1}^{n}y_{i}\cdot p_{i}\left(t_{i}\right) \end{array}$$ (2)

The three terms denote the vehicle fixed costs, variable routing costs, and time window penalty costs $p_{i}(t_{i})$, respectively. Given a delay threshold Δt, $p_{i}(t_{i})$ increases linearly for delays within Δt and grows exponentially beyond it, where μ is the unit time window penalty cost. $p_{i}(t_{i})$ is defined as follows:


$$\begin{array}{c} p_{i}\left(t_{i}\right)=\left\{ \begin{array}{cc} 0, & a_{i}\leq l_{i},\forall i\in N\\ \mu \cdot \left(a_{i}-l_{i}\right), & l_{i}<a_{i}\leq l_{i}+\Delta t,\forall i\in N\\ \mu^{\left[a_{i}-\left(l_{i}+\Delta t\right)\right]}+\mu \cdot \Delta t, & a_{i}>l_{i}+\Delta t,\forall i\in N \end{array}\right. \end{array}$$ (3)

#### 3.3.3 s.t.


$$\begin{array}{c} y_{i}=\left\{ \begin{array}{cc} 1, & L_{i}\geq \theta \\ 0, & L_{i}<\theta \end{array}\right.\forall i\in N \end{array}$$ (4)

$$\begin{array}{c} \sum_{k=1}^{m}\sum_{j=0}^{n}x_{ij}^{k}=y_{i},\forall i\in N^{\prime } \end{array}$$ (5)

$$\begin{array}{c} \sum_{k=1}^{m}\sum_{i=0}^{n}x_{ij}^{k}=y_{j},\forall j\in N^{\prime } \end{array}$$ (6)

$$\begin{array}{c} \sum_{i=1}^{n}q_{i}\cdot y_{i}\leq Q\cdot \sum_{k=1}^{m}u_{k},\forall k\in K,\forall i\in N^{\prime } \end{array}$$ (7)

$$\begin{array}{c} a_{i}+s_{i}+t_{ij}\cdot x_{ij}\leq a_{j}+M\cdot \left(1-x_{ij}^{k}\right),\forall i\in N^{\prime },\forall j\in N^{\prime },\forall k\in K \end{array}$$ (8)

$$\begin{array}{c} t_{ij}=d_{ij}/v \end{array}$$ (9)

$$\begin{array}{c} e_{i}\cdot y_{i}\leq a_{i}\leq l_{i}\cdot y_{i},\forall i\in N \end{array}$$ (10)

$$\begin{array}{c} \sum_{i=1}^{n}x_{0i}^{k}=u_{k},\forall i\in N^{\prime },\forall k\in K \end{array}$$ (11)

$$\begin{array}{c} u_{i}-u_{j}+N^{\prime }\cdot x_{ij}^{k}\leq N^{\prime }-1,\forall k\in K,\forall i\in N^{\prime },j\in N^{\prime },i\text{≠}j \end{array}$$ (12)

Eq (4) determines whether a customer is included in the delivery service; Eqs (5) and (6) ensure that only selected customers are included in the vehicle’s routes; Eq (7) requires that the total demand of any selected customer set does not exceed the maximum load capacity of the assigned vehicle; Eqs (8)–(10) enforce that the vehicle’s arrival time at each selected customer falls within its respective allowed time windows, where *M* denotes a sufficiently large positive number; Eq (11) requires all delivery vehicles to depart from and return to the depot; Eq (12) preserves the continuity of vehicle travel routes and eliminates subtours, where $u_{i}$ is an auxiliary variable representing the sequence of selected customers *i* in the vehicle’s route.

## 4 Algorithm design

In the field of instant delivery, the VRP-CLM is a variant of VRP, which is a typical NP-hard combinatorial optimization problem. NP-hard problems typically involve extremely high computational complexity, making exact optimal solutions rarely obtainable.

The Genetic Algorithm (GA) was first proposed by Holland [36]. With its global search capability, inherent parallelism, and high compatibility for hybrid integration with other heuristics, GA has become one of the most effective methods for solving such complex routing problems. Previous studies have verified the effectiveness and superiority of GA in this domain [37].

Compared with the TGA, the IGA proposed in this study includes the following key enhancements:

1. **Initial solution construction using the minimum-cost nearest neighbor algorithm (NNC)**

Unlike the traditional initialization method used in TGA, the NNC constructs routes by iteratively selecting the node with the lowest insertion cost. It checks constraints such as time windows and vehicle capacity at each step to ensure a high-quality initial solution.

2. **Selection operator based on stochastic universal sampling (SUS)**

Compared with the roulette wheel selection mechanism, the equidistant deterministic sampling of SUS ensures that the number of selections for each individual is proportional to its fitness. Furthermore, integrating the Generation Gap (GGAP) strategy preserves population diversity and avoids premature convergence.

- 3. **Integration of a greedy-strategy-incorporated local search algorithm (LSA)**

The LSA conducts neighborhood searches on the current solution through removal-reinsertion operations, which strengthens the algorithm’s ability to escape local optima. Unlike classical neighborhood search methods (e.g., 2-opt, 3-opt) that generate solutions via fixed patterns such as customer swapping, these operations are based on the comprehensive correlation between customers. During reinsertion, both constraints and global cost optimization are considered, leading to more flexible neighborhood generation and further improving the local optima avoidance capability.

### 4.1 Encoding

This algorithm uses natural number encoding (i.e., the chromosome). The length of the chromosome is defined as N+K−1, where *N* is the number of customers and *K* is the number of vehicles used. Values greater than *N* serve as route separators to distinguish different vehicle routes. Customers before a separator are assigned to one vehicle, and those after the separator are assigned to the next vehicle.

### 4.2 Population initialization

The initial solution is constructed using the NNC, with the detailed steps as follows

Step 1: Initialization

Set the vehicle departure time and dispatch the first vehicle from the distribution center.

Step 2: Node selection

Calculate the insertion cost and select the starting node with the minimum cost. If the node satisfies constraints such as time window and vehicle capacity, add it to the current route.

Step 3: Route construction

Iteratively select the next customer according to the rule in Step 2, and check whether the total demand of the current route is less than the vehicle’s load capacity. If the total demand reaches or exceeds the vehicle’s load capacity, increment the vehicle count and start constructing a new route.

Step 4: Termination condition

The algorithm terminates when all eligible customers have been assigned to routes, and all routes are allocated to vehicles. The insertion cost $C_{ij}$ is defined as the weighted sum of three components: travel time $t_{ij}$ between nodes *i* and *j*, the time window proximity $P_{ij}$ between nodes *i* and *j*, and the time window urgency $U_{j}$ of customer node *j*. Its calculation is given in Eq (13). Here:

$P_{ij}$ denotes the difference between the service start time at subsequent node *j* and the service completion time at previous node *i* (see Eq (14)); $U_{j}$ denotes the difference between the latest allowable service time and the earliest allowable service time at node *j* (see Eq (15)).

The specific formulas are as follows:


$$\begin{array}{c} C_{ij}=\omega_{1}\cdot t_{ij}+\omega_{2}\cdot P_{ij}+\omega_{3}\cdot U_{j} \end{array}$$ (13)

$$\begin{array}{c} P_{ij}=a_{j}-(a_{i}+s_{i}) \end{array}$$ (14)

$$\begin{array}{c} U_{j}=l_{j}-(a_{i}+s_{i}+t_{ij}) \end{array}$$ (15)

Where: $\omega_{1}$, $\omega_{2}$, $\omega_{3}$ are weight coefficients satisfying $\omega_{1}+\omega_{2}+\omega_{3}=1$; $a_{i}$ is the service start time at node *i*; $s_{i}$ is the service duration at node *i*; and $l_{j}$ is the latest allowable service time at node *j*.

### 4.3 Construction of the fitness function

The fitness function in this study is defined as the reciprocal of the objective function, which can be expressed as:


$$\begin{array}{c} f(i)=1/T_{c}(i) \end{array}$$ (16)

where *f*(*i*) denotes the fitness value of the *i*-th chromosome, and $T_{c}(i)$ represents the total delivery cost corresponding to the *i*-th chromosome.

### 4.4 Selection operator

The selection operator employs the SUS strategy, and its detailed procedure is described as follows:

Assume there are 6 individuals with fitness values {6.76, 1.2, 8.49, 2.46, 3.19, 4.25}. Let *F* = (6.76 + 1.2 + 8.49 + 2.46 + 3.19 + 4.25) = 26.35 denote the total fitness, and *N* represent the number of individuals to be selected.

Step 1: Calculate the spacing between pointers, denoted as *P* = *F* / *N* = 26.35 / 6 = 4.39.
Step 2: Assume a random starting position of the pointer is generated $S_{start}=1.50$ (a random number within the range of 0 to *P*).
Step 3: Calculate the position of each pointer as $P_{pointers}=S_{start}+i\times P$, i=(0,1,…,N−1). The resulting pointer positions are (1.50, 5.89, 10.28, 14.67, 19.06, 23.45).
Step 4: Select individuals according to the positions of the pointers; the selected indices are (1, 1, 3, 3, 5, 6).

**Fig 1:**
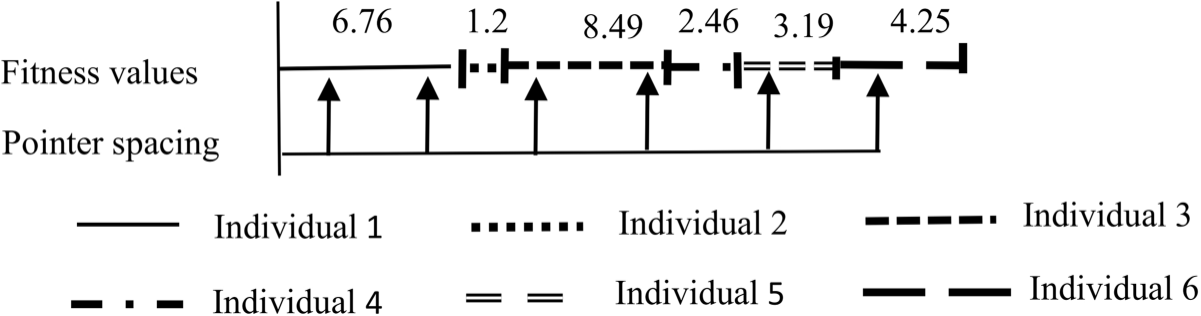
Schematic diagram of the SUS selection strategy.

The detailed selection process is illustrated in Fig 1.

To enhance population iteration efficiency, the GGAP strategy is integrated simultaneously. Specifically, in each generation, a fraction of (1−GGAP) of parent individuals with highest fitness (screened via fitness ranking) is retained. For the remaining fraction GGAP, parent individuals are selected through SUS, and offspring are generated through crossover and mutation. These retained parents and new offspring are then combined to form the next-generation population.

### 4.5 Crossover operator

This algorithm employs the order crossover operator. The detailed procedure is described as follows:

Assume there are two parent chromosomes, denoted as *R*_1_ and *R*_2_, which represent two feasible solutions:

*R*_1_ = {1, 3, 6, 2, 4, 5, 8, 7, 9},
*R*_2_ = {8, 7, 5, 3, 9, 4, 1, 6, 2}.

**Fig 2:**
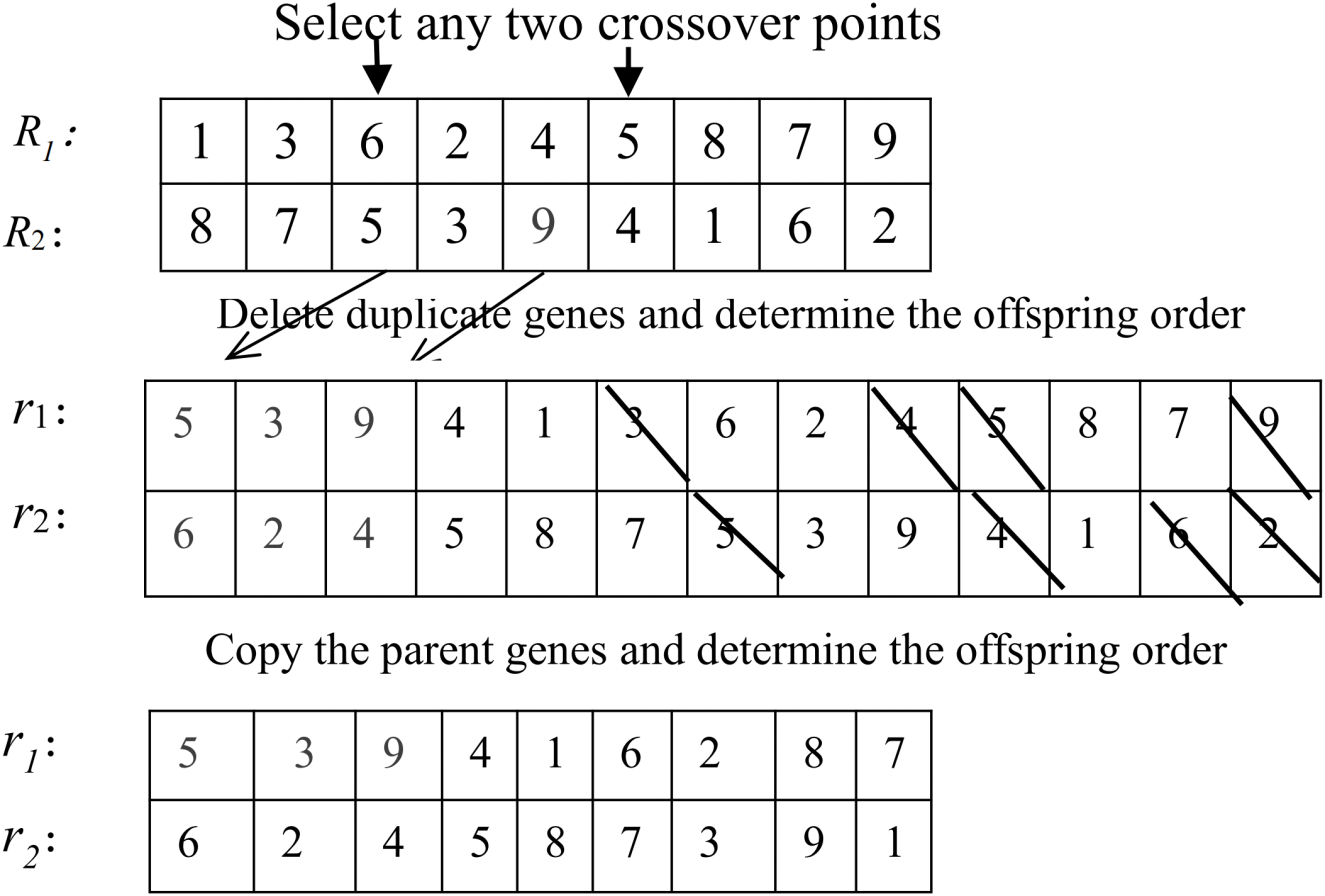
Schematic diagram of crossover operator operation.

Two crossover points are randomly selected at positions 3 and 7. The crossover process to generate offspring *r*_1_ and *r*_2_ is illustrated in Fig 2.

### 4.6 Mutation operator

This algorithm implements mutation by randomly reversing the gene segments between two positions. The detailed procedure is described as follows:

Assume a chromosome *P*_1_ = {2, 5, 6, 1, 4, 3} and the mutation probability $p_{m}$ is set to 0.05. If a randomly generated number exceeds $p_{m}$, the current chromosome remains unmutated; otherwise, mutation is executed.

Suppose a random permutation *R* = {4, 3, 2, 1, 5, 6} is generated. Based on the mutation probability, the 2nd and 5th gene positions of chromosome *P*_1_ undergo reversal mutation, following the order specified in random permutation *R*. This results in a new chromosome *P*_2_ = {2, 4, 6, 1, 5, 3}. A random permutation *R* = {4, 3, 2, 1, 5, 6} is generated. According to the mutation probability, the 2nd and 5th gene positions of chromosome *P*_1_ are reversed to produce a new chromosome *P*_2_ = {2, 4, 6, 1, 5, 3}.

**Fig 3:**
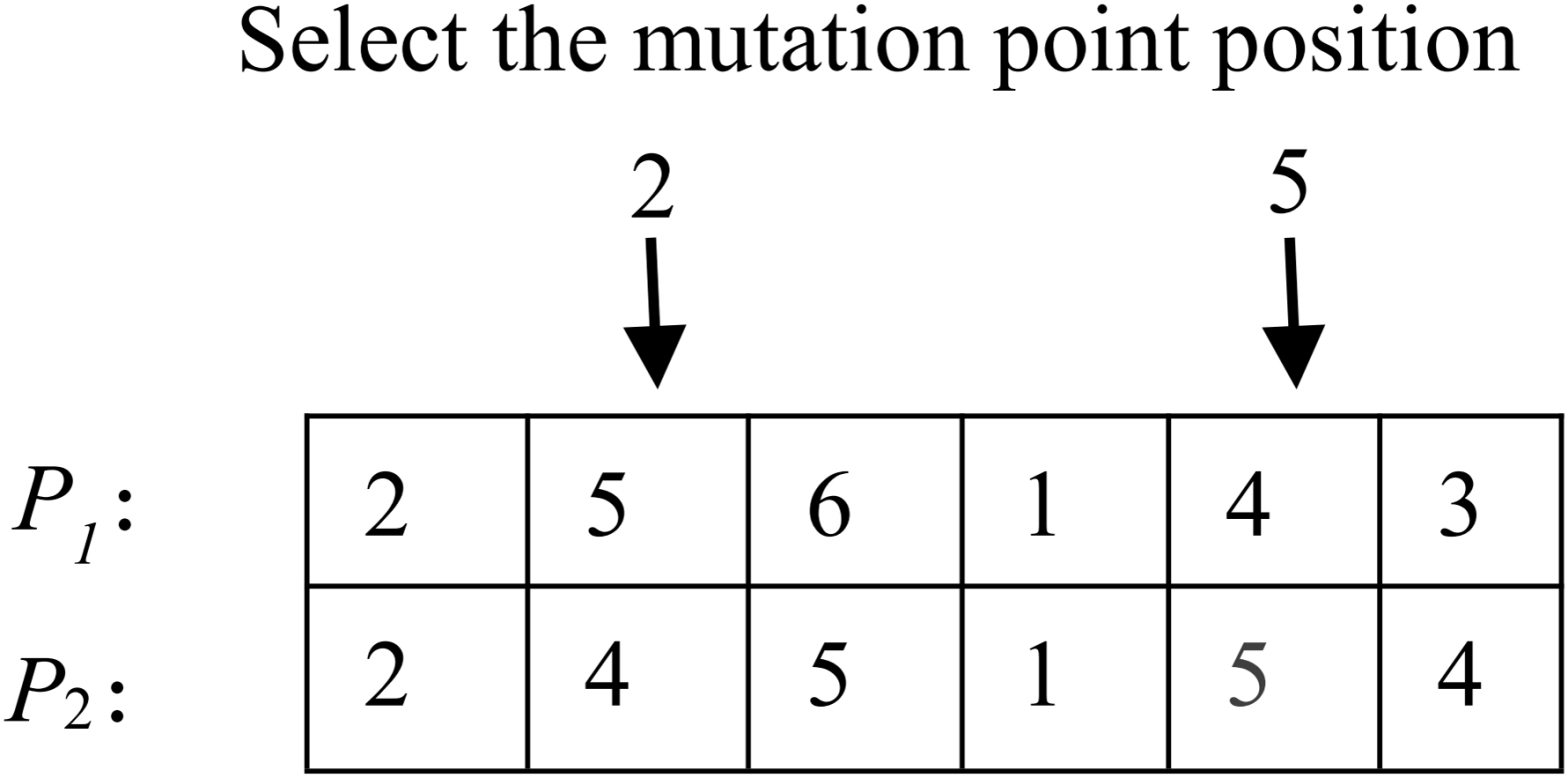
Schematic diagram of mutation operator operation.

The specific operation is illustrated in Fig 3.

### 4.7 Local search algorithm operator

The local search operator employs the LSA strategy. The detailed procedure is as follows:

Step 1: Initialization

Select an initial solution as the starting point and obtain the corresponding route information.

Step 2: Customer removal operation

Based on a comprehensive correlation strategy, the algorithm removes a certain number of customers from the current route to generate a new route. The removal process consists of two phases:

1. Randomly select one customer as the initial candidate for removal.
2. Execute the iterative removal loop: calculate the comprehensive correlation between already removed customers and remaining customers, sort the remaining customers in descending order of correlation, and select subsequent customers for removal in that order. Customers with higher comprehensive correlation have a greater probability of being selected.

Comprehensive correlation $r_{ij}^{\prime }$ consists of distance correlation $c_{ij}$ and route correlation $v_{ij}$, computed as:


$$\begin{array}{c} r_{ij}^{\prime }=\frac{1}{\alpha_{1}\cdot c_{ij}+\alpha_{2}\cdot v_{ij}} \end{array}$$ (17)

where $\alpha_{1}$ (weight for distance correlation) and $\alpha_{2}$ (weight for route correlation) are weight coefficients satisfying $\alpha_{1}+\alpha_{2}=1$. $c_{ij}$: Reflects the spatial correlation between customers *i* and *j*, calculated as:


$$\begin{array}{c} c_{ij}=d_{ij}/md_{i} \end{array}$$ (18)

Here, $d_{ij}$ denotes the distance between customer *i* and customer *j*, and $md_{i}$ represents the maximum distance from the customer *i* to all other customers. $v_{ij}$: Indicates whether customers *i* and *j* are on the same route, defined as a binary variable:


$$\begin{array}{c} v_{ij}=\left\{ \begin{array}{cc} 1 & \text{if customers }i\text{ and }j\text{ are on the same route;}\\ 0 & \text{otherwise.} \end{array}\right. \end{array}$$

In instant delivery scenarios, vehicle travel distance is directly linked to variable routing costs, while the rationality of route structure impacts delivery efficiency and service quality. This study deems both factors equally critical for optimizing delivery routes and achieving cost reduction goals.

Step 3: Customer reinsertion operation

For each removed customer, the Cheapest Insertion Heuristic is used to find its optimal reinsertion position. The process involves two key steps:

1. Calculate the distance increment Δd for each potential insertion point between nodes in the existing routes. The distance increment is determined by comparing the total route distance before and after inserting the customer, using the formula:

$$\begin{array}{c} \Delta d=d_{ik}+d_{kj}-d_{ij} \end{array}$$ (19)

Here, $d_{ij}$, $d_{ik}$, and $d_{kj}$ represent the distances between customer *i* and *j*, customer *i* and *k*, and customer *k* and *j*, respectively.

2. Select the insertion point that minimizes the total distance increment while satisfying both time window constraints and vehicle capacity constraints. If no valid potential insertion exists, assign the customer to a new vehicle and insert it at the start of the new route.

Step 4: Cost recalculation, comparison, and update

Calculate the total delivery cost of the new route after reinsertion. If the cost of the new route is lower than the original cost, update the current solution to the new route; otherwise, keep the original solution.

Step 5: Output the optimal solution

When the predefined number of iterations or other stopping criteria are met, output the optimal solution.

### 4.8 Offspring reinsertion and population update

This algorithm reinserts locally optimized offspring into the main population, ensuring the population includes solutions improved by local search. This strategy maintains population diversity while preventing premature convergence.

The specific strategy is as follows: First, parent individuals are sorted by fitness in ascending order (from lowest to highest). The first *n* parent individuals with the lowest fitness are retained, where n=parent population size−offspring population size. Second, all offspring individuals are added to these selected parent individuals, ultimately forming a new combined population.

### 4.9 Handling duplicate individuals and population replenishment

During each generation of iteration, the algorithm identifies and removes duplicate individuals. To maintain a constant main population size, new individuals are generated randomly to replenish the number of individuals removed.

### 4.10 Termination condition

This algorithm adopts the maximum number of iterations as its termination condition.

## 5 Case analysis

### 5.1 Source of experimental case and model parameter settings

Drawing on the case study by Yu et al. [11], this study adopts the instant delivery platform scenario for validation. The effectiveness of the VRP-CLM model and the IGA is verified by conducting instant delivery services for 36 customers. In this test instance, customers have been classified into different categories, and all specific parameter settings are kept consistent with those reported in the original literature, thereby ensuring the authenticity and reliability of the experimental results. Furthermore, to evaluate the scalability and robustness of the proposed model and algorithm in more complex scenarios, large-scale computational examples are designed and verified in the large-scale supplementary experiments subsection.

All experiments are implemented in MATLAB 2021b. The computer configuration is as follows: Windows (Chinese version), 13th Gen Intel(R) Core(TM) i9-13900H (2.60 GHz), and 32.0 GB RAM.

### 5.2 Calculation of customer loss values and definition of customer loss range

There are various methods for constructing the customer loss mechanism evaluation function, including the Analytic Hierarchy Process, Entropy Method, AHP-Entropy Weight Method, and Principal Component Analysis. Among these, the AHP-Entropy Weight Method offers distinct advantages: it not only allows subjective weighting based on expert experience to accurately capture the implicit logic of customer value evaluation, but also conducts objective weighting according to the dispersion degree of indicator data, thus avoiding excessive bias from subjective preferences [38]. By integrating professional expertise with objective data patterns, this hybrid approach makes the customer loss mechanism evaluation function both scientifically sound and practically applicable. Detailed calculation procedures for the AHP-Entropy Weight Method can be found in Boukrouh et al. [38].

Since the customer loss mechanism evaluation function involves the comprehensive calculation of multi-dimensional indicators (e.g., customer value, time window width) with distinct units and scales, direct computation would cause deviations. To resolve this issue, this study adopts the min-max normalization method to process the raw data. By scaling all indicator values into the interval [0, 1], it eliminates dimensional influences and ensures comparability across indicators.

Accordingly, we construct the customer loss mechanism evaluation function using the AHP-Entropy Weight Method, with its specific formulation given as follows:


$$\begin{array}{c} L_{i}^{\prime }=0.5236\cdot c_{ve}(i{)}^{\prime }+0.1749\cdot c_{tw}(i{)}^{\prime }+0.1086\cdot c_{dd}(i{)}^{\prime }+0.1902\cdot c_{dv}(i{)}^{\prime } \end{array}$$ (20)

In Equation (20), $L_{i}^{\prime }$, $c_{ve}(i{)}^{\prime }$, $c_{tw}(i{)}^{\prime }$, $c_{dd}(i{)}^{\prime }$ and $c_{dv}(i{)}^{\prime }$ denote the normalized values of customer value, time window width, distance from the depot, and demand volume for the *i* -th customer, respectively. The corresponding coefficients represent the weights assigned to these influencing factors. The calculated results of the customer loss values $L_{i}^{\prime }$, along with the values of $c_{ve}(i{)}^{\prime }$, $c_{tw}(i{)}^{\prime }$, $c_{dd}(i{)}^{\prime }$, $c_{dv}(i{)}^{\prime }$, are summarized in Table 2.

**Table 2:** Normalized values of customer loss impact factors and customer loss values.

IDs	$c_{ve}^{\prime }$	$c_{tw}^{\prime }$	$c_{dd}^{\prime }$	$c_{dv}^{\prime }$	$L_{i}^{\prime }$	IDs	$c_{ve}^{\prime }$	$c_{tw}^{\prime }$	$c_{dd}^{\prime }$	$c_{dv}^{\prime }$	$L_{i}^{\prime }$
1	0.0000	0.8095	0.0000	0.3556	0.2093	19	0.5000	0.2857	0.2071	0.2931	0.3914
2	0.0000	0.6190	0.1314	0.0108	0.1246	20	1.0000	0.2857	0.3682	0.8345	0.7750
3	0.0000	0.6667	0.3782	0.0459	0.1664	21	1.0000	0.3333	0.8903	0.6884	0.8122
4	1.0000	0.4762	0.7678	1.0000	0.8832	22	0.5000	0.0952	0.4721	0.0143	0.3338
5	0.0000	0.6190	0.0751	0.1607	0.1470	23	0.5000	0.5714	0.3117	0.0721	0.4107
6	0.0000	0.8095	0.7876	0.2497	0.2746	24	1.0000	0.3333	0.5964	0.2002	0.6874
7	0.0000	0.7143	0.5431	0.0271	0.1891	25	1.0000	0.2381	0.2386	0.4090	0.6716
8	1.0000	0.3810	0.6109	0.1493	0.6876	26	0.5000	0.3810	0.4570	0.4218	0.3801
9	0.0000	0.9048	0.1030	0.1048	0.1894	27	0.5000	0.3810	0.4465	0.0316	0.3843
10	0.5000	0.2857	0.5898	0.1736	0.4102	28	0.0000	0.6667	0.8704	0.6072	0.3266
11	0.5000	0.4762	0.7042	0.0970	0.4413	29	0.5000	0.5714	0.0673	0.1596	0.4008
12	0.5000	0.5238	0.0608	0.1377	0.3876	30	0.0000	0.7143	0.2157	0.0000	0.1484
13	0.0000	0.8571	0.1555	0.2059	0.2060	31	1.0000	0.0000	0.7664	0.5571	0.7154
14	0.0000	0.8571	0.7698	0.2860	0.2879	32	0.0000	0.4286	1.0000	0.0446	0.1920
15	0.5000	0.6190	0.1278	0.0946	0.4033	33	0.0000	0.7143	0.9372	0.1746	0.2599
16	0.0000	0.6667	0.9831	0.2553	0.2719	34	0.0000	0.8095	0.4440	0.2449	0.2364
17	0.5000	0.5714	0.0279	0.0808	0.3815	35	0.0000	1.0000	0.3848	0.0712	0.2303
18	0.5000	0.2857	0.3470	0.2931	0.4065	36	0.0000	0.7619	0.0679	0.0271	0.1458

According to the proportional exclusion method developed in the subsection titled Construction of the customer loss mechanism evaluation function, this section applies an allowed loss proportion of no more than 5% and 10% to determine the specific customer loss thresholds and the corresponding sets of excluded customers. To ensure the actual number of discarded customers does not exceed the target proportion, the excluded count is strictly rounded down. Based on the sorted $L_{i}^{\prime }$ values in Table 2, the specific schemes are defined as follows:

Under the 5% maximum allowed loss proportion, 5% of 36 customers is 1.8, which is rounded down to 1 customer. The customer with the lowest customer loss value is ID 2 ($L_{2}^{\prime }=0.1246$), while the next lowest is ID 36 ($L_{36}^{\prime }=0.1458$). Therefore, the threshold is set to $\theta_{5\%}=0.05$. Consequently, customer 2 is excluded from service, keeping the actual allowed loss proportion at 2.78%, which is within the 5% limit.

Under the 10% maximum allowed loss proportion, 10% of 36 customers is 3.6, which is rounded down to 3 customers. The three customers with the lowest customer loss values are IDs 2, 36, and 5 ($L_{35}^{\prime }=0.1470$), while the fourth lowest is ID 30 ($L_{30}^{\prime }=0.1484$). Consequently, the threshold is set to $\theta_{10\%}=0.06$. Customers 2, 36, and 5 are excluded, keeping the actual allowed loss proportion at 8.33%, within the 10% limit.

### 5.3 Comparative analysis of algorithms and schemes

This study designs two computational schemes for comparison:

Scheme 1:

To validate the performance of the IGA, comparative experiments are conducted using the IGA, TGA, and TS for delivery schemes without the customer loss mechanism.

Scheme 2:

To verify the effectiveness of the proposed customer loss mechanism, the VRP-CLM model is evaluated under two maximum allowed loss proportion scenarios: a cap of 5% (excluding 1 customer) and a cap of 10% (excluding 3 customers). This setup aims to analyze how different scales of customer loss impact cost optimization.

The key parameter settings for the experiments are presented in Table 3.

**Table 3:** Key Parameter Settings in Experiments.

Parameter	Description	Value
*MAXGEN*	Maximum number of iterations	300
*NIND*	Population size (IGA and TGA)	100
$p_{c}$	Crossover probability	0.9
$p_{m}$	Mutation probability	0.05
*GGAP*	Generation gap (for IGA)	0.9
*bLength*	Tabu list length (for TS)	20
λ	Tabu search coefficient	0.015

#### 5.3.1 Algorithm performance analysis.

To evaluate the performance of the IGA against the TGA and TS, this study conducts a comparative analysis from three dimensions: convergence speed, solution quality, and robustness. The convergence speed and runtime of the respective algorithms are obtained from the first round of computation. Specifically, the single-run runtime of IGA, TGA, and TS is approximately 60 seconds, 15 seconds, and 10 seconds, respectively.

**Fig 4:**
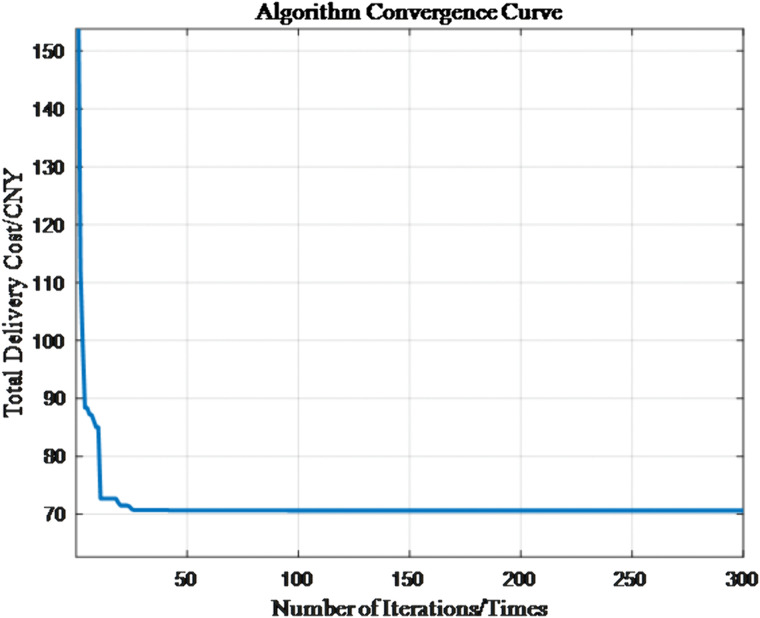
Algorithm convergence curve of IGA.

**Fig 5:**
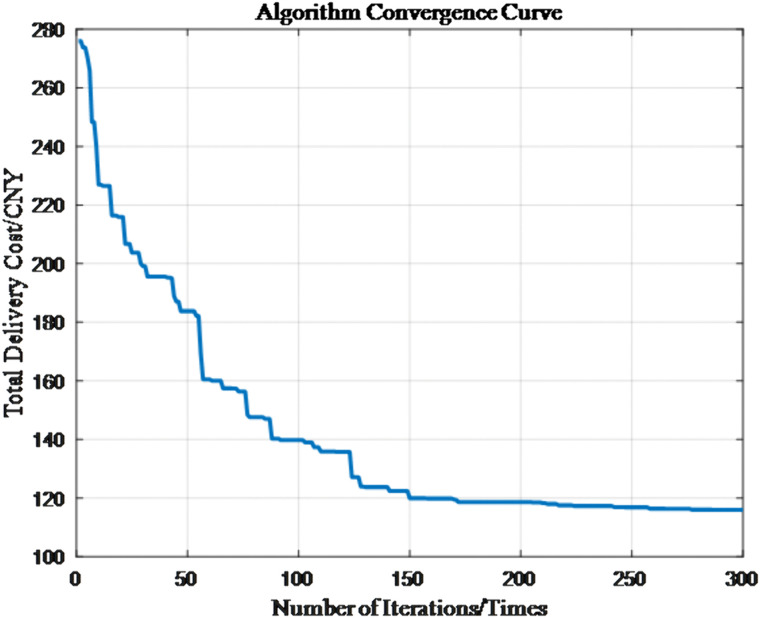
Algorithm convergence curve of TGA.

**Fig 6:**
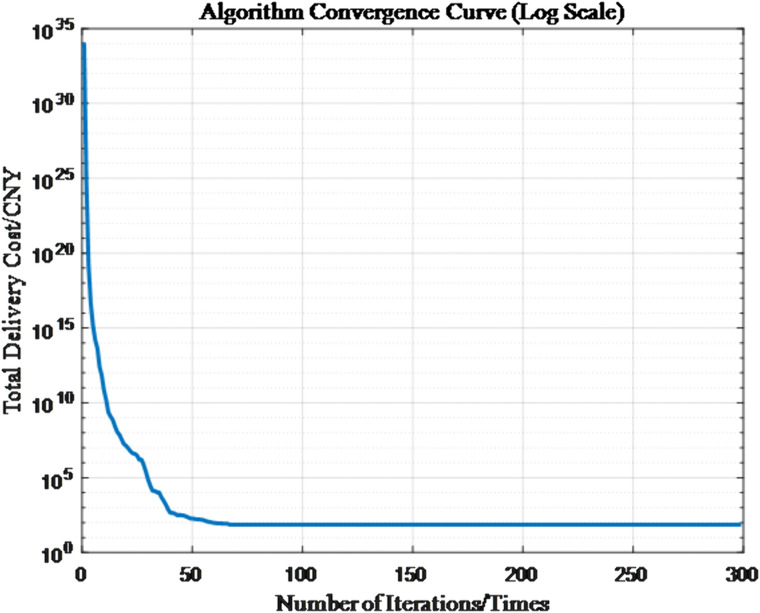
Algorithm convergence curve of TS.

In terms of convergence speed, IGA demonstrates a significant advantage over TGA and TS (see Figs 4–6). As shown in the figures, IGA starts to converge around the 20th generation, whereas TGA and TS require approximately 150th and 50th generations, respectively, to stabilize. The number of iterations required for IGA to converge is only about 2/15 of that for TGA and 2/5 of that for TS, indicating a clear advantage in convergence efficiency.

This result indicates that the IGA can converge to the optimal or a near-optimal solution much faster during optimization, significantly reducing the number of iterations required. It should be noted that while the IGA’s single-run runtime (about 60 seconds) is slightly longer than that of TGA and TS, this difference does not weaken its overall advantage in optimization efficiency. In fact, the fast convergence of the IGA enables it to approach the optimal solution in the early stages of the optimization process and complete the task with fewer iterations. In other words, given the same total computation time, the IGA can obtain high-quality solutions for complex problems more efficiently. In contrast, the TGA and TS, which need more iterations to converge, may be less applicable under constrained computational resources.

From the perspective of solution quality and stability, statistical analysis was conducted on the results of 30 independent runs for the IGA, TGA, and TS (see Table 4). Statistical verification confirmed that all datasets satisfied the assumptions of normality and homogeneity of variance, making them suitable for *t*-tests. The results clearly indicate that the IGA algorithm has significant stability and superior performance in key indicators.

(1) Total delivery cost (TC)

**Table 4:** Optimization Results of the IGA, TGA, and TS with the Objective of Minimizing Total Delivery Cost.

	IGA			TGA			TS		
	TC	TPC	NV	TC	TPC	NV	TC	TPC	NV
	(CNY)	(CNY)	(vehicles)	(CNY)	(CNY)	(vehicles)	(CNY)	(CNY)	(vehicles)
1	70.75	0	5	129.45	0.15	8	70.99	0	5
2	70.46	0	5	115.20	0	8	80.66	6.99	5
3	70.78	0	5	113.75	0.08	8	72.44	0	5
4	70.34	0	5	116.82	0	8	70.68	0	5
5	70.51	0	5	116.58	0.10	8	84.48	8.53	5
6	70.51	0	5	114.61	0	8	85.13	9.62	5
7	70.75	0	5	113.72	0	8	70.65	0	5
8	70.47	0	5	103.88	0	8	72.09	0	5
9	70.50	0	5	107.12	5.81	7	71.05	0	5
10	70.54	0	5	116.44	0	8	71.31	0.06	5
11	70.63	0	5	103.87	3.56	7	70.73	0	5
12	70.75	0	5	101.16	0	7	71.11	0	5
13	70.78	0	5	114.62	0	8	71.36	0	5
14	70.47	0	5	101.17	0.22	7	73.79	0	5
15	70.63	0	5	102.00	0	7	71.49	0	5
16	70.34	0	5	129.22	11.04	8	73.03	0	5
17	70.75	0	5	102.71	1.59	7	70.82	0	5
18	70.66	0	5	115.46	0	8	87.28	11.63	5
19	70.45	0	5	101.84	0	7	71.13	0	5
20	70.57	0	5	107.34	6.99	7	71.89	0	5
21	70.41	0	5	115.40	0.05	8	71.20	0	5
22	70.75	0	5	115.71	0.06	8	79.02	5.72	5
23	70.46	0	5	103.95	0.92	7	71.64	0	5
24	70.46	0	5	101.22	0	7	70.77	0	5
25	70.75	0	5	101.12	0	7	76.69	3.47	5
26	70.34	0	5	115.74	0	8	70.59	0	5
27	70.45	0	5	102.28	0	7	72.60	0	5
28	70.48	0	5	121.17	5.04	8	71.03	0	5
29	70.75	0	5	106.45	4.48	8	70.94	0	5
30	70.63	0	5	110.71	8.29	7	73.04	0	5
avg	70.57	0	5	110.69	1.61	7.53	73.65	1.53	5
*s*	0.15	0	0	8.11	2.98	0.49	4.73	3.34	0

For the total delivery cost, statistical results show that the IGA achieves outstanding performance in cost control. Specifically, its average total delivery cost (avg) is only 70.57 CNY, which is 40.12 CNY lower than that of the TGA (110.69 CNY), representing a 36.25% reduction, and 3.08 CNY lower than that of the TS (73.65 CNY), a 4.18% reduction. Moreover, the standard deviation (*s*) of the IGA’s total delivery cost is merely 0.15 CNY, far smaller than 8.11 CNY for the TGA and 4.73 CNY for the TS, highlighting the high stability of its solutions.

To further explore the sources of cost savings, we analyzed the number of vehicles used. On average, the IGA uses 2.53 fewer delivery vehicles than the TGA. Cost savings from reduced vehicle usage account for 75.67% of the total delivery cost reduction, while savings from route optimization make up the remaining 24.33%. This indicates that the IGA effectively improves both resource allocation and route planning.

Further statistical tests confirm that the difference in average total delivery cost between the TGA and IGA is highly statistically significant (*p* < 0.001), and the difference between the TS and IGA is also significant (*p* < 0.001). These results further validate IGA’s clear advantage in total delivery cost control.

(2) Time window penalty costs (TPC)

In terms of time window penalty cost, 30 independent runs of the IGA yield a consistently zero penalty cost, achieving full compliance with no time window breaches. This demonstrates that the IGA provides exceptional stability in controlling time window penalty expenditure. In contrast, the average time window penalty costs from 30 independent runs are 1.61 CNY for the TGA and 1.53 CNY for the TS. Their standard deviations are 2.98 CNY and 3.34 CNY, respectively, with time window violation occurrence rates of 50.00% and 23.33%. These results reveal considerable performance fluctuations. This comparison fully confirms the IGA’s reliable performance in mitigating time window violation risks.

Statistical tests further validate this conclusion: the difference in average time window penalty cost between the TGA and IGA is highly statistically significant (*p* < 0.001), and the difference between TS and IGA is also significant (*p* < 0.001).

(3) Number of vehicles used (NV)

In terms of the number of vehicles used, both the IGA and TS maintain a stable average of 5 vehicles, with a standard deviation of 0. By contrast, the TGA yields an average of 7.53 vehicles, with a standard deviation of 4.73. This demonstrates that IGA also outperforms traditional algorithms in the stability of resource allocation efficiency.

(4) Comprehensive Conclusion

Overall, the IGA exhibits dual advantages: the lowest mean value and the smallest fluctuation across three core dimensions—total delivery cost control, violation risk avoidance, and vehicle resource allocation. Its advantages are not only statistically significant but also translate into substantial practical benefits in scenarios. Specifically, it achieves an average cost reduction of 36.3% compared to the TGA, and reduces the violation rates from 23.33%–50.00% down to 0. These results confirm the stability and reliability of the IGA.

Having verified the superiority of the IGA in optimizing delivery routes, the comparative analysis of VRP-CLM subsection further validates the effectiveness of the proposed VRP-CLM model by comparing it with a scheme without the customer loss mechanism.

#### 5.3.2 Comparative analysis of VRP-CLM.

As shown in Table 4), the average total delivery cost of Scheme 1 (calculated using the IGA across 30 runs) is 70.57 CNY, of which the vehicle fixed costs are 60 CNY—accounting for 85.02% of the total delivery cost. This indicates that when vehicle fixed costs account for a large proportion of the total delivery cost, reducing the fleet size is key to lowering the total delivery cost. This finding also provides an important cost structure basis for subsequent strategy implementation.

Within Scheme 2, the delivery scenario under a maximum allowed loss proportion of 5% (excluding only 1 customer, accounting for merely 2.78% of the total) is first evaluated. Repeated IGA iterations show that the total delivery cost stabilizes steadily at around 70.41 CNY, indicating that such a low allowed loss proportion cannot yield a satisfactory cost-optimization outcome. The essential reason is that, in this small-scale customer network, the 5% loss threshold is too low to reach the system’s critical resource bottleneck. Excluding only one node with marginal resource consumption neither reduces the required fleet size (which remains fixed at 5 vehicles) nor restructures the existing routing framework, thus failing to cut down the dominant vehicle fixed costs.

By contrast, under the scenario with a maximum allowed loss proportion of 10% (where the actual loss is 8.33%), the IGA successfully identifies a satisfactory solution that substantially reduces costs. The specific delivery scheme is presented in Table 5. By excluding a small fraction of customers (8.33% of the total), the demands of the remaining 91.67% of customers are effectively met within the predefined time windows. With zero time window violations, the total delivery cost is significantly reduced from 70.57 CNY in Scheme 1 to 58.44 CNY, a decrease of 17.18%. Notably, the reduction in vehicle fixed costs—achieved by decreasing the fleet size from 5 (Scheme 1) to 4 (Scheme 2). This verifies that excluding three key resource-intensive customers is sufficient to restructure overall delivery routes and free up an entire vehicle’s capacity.

**Table 5:** Distribution plan for plan 2.

Total delivery cost	Delivery route	Constraint-violating customers
58.44 CNY	0-33-7-18-8-27-23-15-13-0	/
	0-11-22-21-32-16-28-6-14-0	/
	0-31-10-26-20-19-17-35-34-0	/
	0-4-24-25-12-1-29-9-0	/

Specifically, the scenario with a maximum allowed loss proportion of 10% within Scheme 2 significantly reduces the total delivery cost while excluding only a small fraction of customers (8.33%) and fully meeting the service needs of the remaining customers. This indicates that by proactively optimizing delivery resource allocation—specifically, by strategically excluding marginal demands at a scale sufficient to reduce vehicle usage—enterprises can not only safeguard the core demands of most customers but also effectively control costs and substantially improve operational efficiency. This result effectively verifies the following conclusion: For instant delivery services, when delivery resources and customer demands are mismatched, proactively excluding hard-to-serve customers is superior to passively losing customers. However, as demonstrated by the negligible effect of the scenario with a maximum 5% allowed loss proportion, this proactive loss must reach a critical scale—sufficient to eliminate entire vehicle routes—to yield significant operational benefits; otherwise, its practical value remains limited. At the same time, it also indirectly illustrates that if enterprises blindly pursue full demand coverage under resource constraints, attempting to meet the needs of a very small proportion of customers may trigger a sharp rise in total delivery costs, which is counterproductive to overall efficiency improvement.

### 5.4 Large-scale supplementary experiments

To further validate the effectiveness of the customer loss mechanism and the VRP-CLM model in large-scale, highly constrained instant delivery scenarios, this section conducts supplementary experiments using the Solomon benchmark instance C101, which is solved via the IGA. This instance consists of one distribution depot and 100 customer nodes, characterized by clustered customer distributions and tight time window constraints. These features closely align with real-world instant delivery conditions during peak hours.

#### 5.4.1 Experimental design.

To reflect the real-world characteristics of instant delivery—short delivery distances, high urgency, and narrow time windows—this section linearly rescales the original Solomon C101 instance data. This transformation compresses the original parameters into a realistic instant delivery scope (expressing time in minutes and distance in kilometers) while fully preserving the relative tightness of the time windows and the clustered distribution from the original instance. Specifically, the instance is rescaled to align with a typical 3 km service radius and a 30-minute scheduling horizon. The specific transformation rules are as follows:

1. **Spatial coordinate scaling**

To map the original spatial range of [0, 100] (normalized coordinates) to a typical 3 km service radius for instant delivery, we define a spatial scaling coefficient κ. The scaled coordinates are computed by $x_{i}^{\prime }=x_{i}\times \kappa$ and $y_{i}^{\prime }=y_{i}\times \kappa$, where $\left(x_{i},y_{i}\right)$ and $\left(x_{i}^{\prime },y_{i}^{\prime }\right)$ represent the original and scaled coordinates, respectively.

2. **Linear mapping of time window width**

Let the original time window width of customer *i* in C101 be $tw_{i}$, and let the range of the original time window widths are $\left[tw_{\min},tw_{\max}\right]$. The target range of time window widths for the instant delivery scenario is set as $\left[tw_{\min}^{\prime },tw_{\max}^{\prime }\right]$. The mapped time window width $tw_{i}^{\prime }$ is computed using the following linear mapping formula:


$$\begin{array}{c} tw_{i}^{\prime }=tw_{\min}^{\prime }+\frac{tw_{i}-tw_{\min}}{tw_{\max}-tw_{\min}}\times (tw_{\max}^{\prime }-tw_{\min}^{\prime }) \end{array}$$ (21)

Based on the mapped width $tw_{i}^{\prime }$ and the latest allowable service time, the time window of each customer is updated accordingly. The time window of the distribution depot is set to [0, 30] minutes.

3. **Other parameter adaptations**

The service time is uniformly set to 1 minute. To eliminate interference from customer value stratification and focus on verifying the impacts of distance, demand volume, and time window width, the customer value is fixed at 1 for all customers. The vehicle load capacity retains the original setting from Solomon (1987) at 200. All other algorithm parameters (e.g., population size, maximum number of iterations), vehicle speed, and vehicle fixed cost remain consistent with those in the preceding section.

4. **Comparative schemes**

To validate the effectiveness of the customer loss mechanism, the following schemes are designed:

- Baseline scheme (Full Service): all 100 customers are mandatorily served, corresponding to a maximum allowed loss proportion of 0%.
- VRP-CLM Scheme: the proposed model is applied with the maximum allowed loss proportion 5% and 10%, respectively, consistent with the previous small-scale experiments.

Consistent with the small-scale experiments, the performance of the customer loss mechanism in this large-scale scenario is evaluated by comparing the total vehicle travel distance, time window violation rate, and total delivery cost across the three schemes (0%, 5%, and 10% allowed loss proportion).

#### 5.4.2 Experimental results and analysis.

To clearly demonstrate the optimization performance of the VRP-CLM in large-scale instant delivery scenarios, the key delivery indicators under varying maximum allowed loss proportions are compared, as shown in Table 6).

**Table 6:** Comparison of Key Indicators for Large-Scale Instances.

	Full Service Scheme	VRP-CLM Scheme (5% Allowed Loss)	VRP-CLM Scheme (10% Allowed Loss)
Number of Vehicles Used	10	9	8
Total Travel Distance (km)	28.9869	25.236	28.4758
Total Travel Cost (CNY)	131.5948	118.0944	107.3903
Total Penalty Cost (CNY)	0	0	0
Number of Constraint-Violating Customers	0	0	0

As indicated in Table 6), the VRP-CLM significantly reduces cost and enhances efficiency under varying maximum allowed loss proportion. Furthermore, the total delivery cost decreases gradually as the allowed loss proportion increases. When the maximum allowed loss proportion is merely 5%, the model exhibits a pronounced leverage optimization effect: the number of vehicles required decreases significantly from 10 in the full-service scheme to 9, while the total travel distance counter-intuitively drops from 28.99 km to 25.24 km rather than increasing. This phenomenon reveals the inherent drawbacks of the full-service scheme. Under tight time window constraints, accommodating the final 5% of marginal customers—typically located at the spatial edges of clusters or with extremely stringent time windows—compels the system to deploy an additional vehicle and incur ineffective detours. By precisely excluding these low-efficiency, high-cost marginal customers, the VRP-CLM achieves a dual reduction in both vehicle count and travel distance. Consequently, the total delivery cost is reduced by 13.50 CNY (a 10.26% decrease) while maintaining a zero-violation record throughout the process.

As the maximum allowed loss proportion increases 10%, the fleet size is further reduced to 8 vehicles. Notably, consistent with the classic empirical rule in VRP—where reducing the number of vehicles typically comes at the cost of increased per-vehicle mileage—the total travel distance rises to 28.48 km (slightly higher than in the 5% scheme). However, since vehicle fixed costs are significantly higher than variable distance-related costs in this scenario, the vehicle fixed cost savings from eliminating one vehicle fully offset the additional costs incurred by the longer travel distance. Ultimately, the total delivery cost decreases to 107.39 CNY, representing a reduction of 24.20 CNY (18.39%) compared with the full-service scheme.

Furthermore, in the schemes with a maximum allowed loss proportion 5% and 10%, the time window penalty cost and the number of constraint-violating customers remain strictly zero. This verifies that the customer loss mechanism is not an arbitrary rejection mechanism; rather, it demonstrates precise screening and highly efficient adaptive capabilities. The excluded customers are exclusively resource-intensive, high-cost nodes that cause the most severe disruptions to the overall network. By reallocating the freed capacity to focus on the remaining high-priority customers, the system achieves a 100% fulfillment rate even under high-load operations.

In conclusion, experiments on large-scale, highly constrained instances with 100 customers reaffirm the strong robustness and exceptional practical value of the VRP-CLM model. In contrast to passive constraint violations and subsequent overtime penalties, the proactive customer loss mechanism accurately screens out resource-intensive, low-efficiency customers. This effectively eliminates the capacity waste and cost redundancy caused by serving a small share of marginal customers under the full-service paradigm. By sacrificing only a minimal portion of demand (5%–10%), the mechanism achieves a considerable reduction in fleet size (saving 1–2 vehicles) and a significant drop in total delivery costs (up to 18.39%), closely aligning with the practical realities of rigid resource constraints during peak hours in instant delivery.

### 5.5 Sensitivity analysis

In this study, sensitivity analysis is only performed on the real-world case from the core experiments, rather than extended to the large-scale standard instances in the large-scale supplementary experiments subsection. The objective of this sensitivity analysis is to verify the robustness of the indicator weighting mechanism and the customer loss mechanism in practical scenarios. Since the large-scale standard instances are mainly designed to test the computational efficiency and scalability of the proposed algorithm, they do not involve the customer loss mechanism evaluation function or weight calibration. Accordingly, weight sensitivity analysis is not required for these standard instances.

To verify the robustness of the proposed optimization model under the proactive customer loss strategy, this study takes the initial weights of the four indicators ($c_{ve},c_{tw},c_{dd},c_{dv}$) determined by the AHP-entropy method as the benchmark. A multi-dimensional combined perturbation analysis is conducted on each indicator weight across 12 perturbation states (ranging from ±5% to ±25% in 5% increments), yielding a total of 10000 perturbation scenarios.

#### 5.5.1 Weight perturbation and normalization rule.

The weight of a single indicator after perturbation is calculated as follows:


$$\begin{array}{c} \beta_{i}^{\prime }=\beta_{i}\times (1+\delta ) \end{array}$$ (22)

where $\beta_{i}$ is the benchmark weight of each indicator, and δ∈(0,1] represents the relative perturbation amplitude, which controls the magnitude of random variation applied to the weights. Following perturbation, all weights are normalized to satisfy the unit-sum constraint. The final normalized weight is calculated as:


$$\begin{array}{c} {\beta_{i}^{\prime }}^{\prime }=\frac{\beta_{i}^{\prime }}{\sum_{j=1}^{4}\beta_{j}^{\prime }},\;i=1,2,3,4 \end{array}$$ (23)

It has been verified that the perturbed weights still maintain satisfactory consistency.

For each perturbed weight set, the customer loss values are recalculated to determine the customer loss set. The IGA is then adopted to re-solve the optimization model. The variations in customer screening outcomes and total delivery cost are recorded to evaluate the anti-interference ability and robustness of the proposed model and proactive customer loss strategy.

#### 5.5.2 Analysis of weight perturbation results.

Experimental results show that the customer loss set remains completely stable when the weights of the four indicators are perturbed within the range of ±20%. When the perturbation amplitude increases to ±25%, only 100 out of 10,000 combined weight schemes lead to changes in the customer loss set. Notably, all of these 100 altered schemes result in a single identical customer loss set: (2, 5, 30).

Under this altered customer loss set, multiple independent runs of the improved genetic algorithm yield a satisfactory total delivery cost around 59 CNY, with negligible fluctuations compared with the pre-perturbation cost. The slight cost variation is primarily caused by the adjustment of delivery routes, which leads to minor changes in variable routing costs.

The above results fully demonstrate that the customer loss mechanism evaluation function, optimization model, and the proactive customer loss strategy constructed in this study are insensitive to indicator weight perturbations and possess strong anti-interference ability. They maintain high stability in both customer loss results and distribution costs within a wide range of weight fluctuations, proving the model has favorable robustness and reliability.

## 6 Conclusions

(1) This study focuses on the VRP-CLM to address resource-demand mismatches, utilizing the AHP-Entropy Weight Method to construct a customer loss mechanism evaluation function that accurately identifies resource-intensive customers. Both fundamental and large-scale (100-customer) instances validate the model’s effectiveness, revealing a pronounced leverage optimization effect. In the fundamental case, proactively excluding 8.33% of customers reduces total delivery costs by 17.18%. Similarly, large-scale experiments show that at a 5% allowed loss proportion, this exclusion counter-intuitively reduces both fleet size and travel distance; at 10%, vehicle fixed cost savings fully offset the slight distance increase, yielding an 18.39% total delivery cost reduction with zero violations. This demonstrates that the mechanism precisely screens out marginal nodes. Furthermore, sensitivity analysis confirms the model’s robust performance across varying resource tightness, offering flexible strategies for peak-hour delivery.
(2) In terms of algorithm design, the IGA exhibits significant advantages. By incorporating the NNC method for initialization, integrating SUS with GGAP for selection, and designing a greedy-based local search, the IGA effectively escapes local optima. Experimental data strongly confirm the outstanding performance of the IGA: its total delivery cost is reduced by 36.25% compared to the TGA and 4.18% compared to the TS, with zero time window penalty cost. Further statistical tests verify the significance of the IGA’s superiority.
(3) While this study contributes by proposing the VRP-CLM, certain limitations remain. Chief among these is that the current objective function does not consider the explicit costs associated with customer loss, such as revenue loss and churn compensation. While the current model prioritizes short-term operational efficiency, future research should incorporate these explicit customer loss costs into the objective function. Furthermore, incorporating long-term brand value indicators (e.g., customer loyalty) into the customer loss mechanism evaluation function and constructing a multi-objective model balancing short-term costs with long-term brand benefits will enhance the model’s applicability.
